# Side effects of metal-based dental implantology treatment - A review

**DOI:** 10.6026/973206300214048

**Published:** 2025-11-15

**Authors:** Deepesh Kumar Gupta, Shubham Sethi, Anumeha Jha, Ambika Thakur, Khushbu Gupta

**Affiliations:** 1 Department of Oral and Maxillofacial Prosthodontics and Implantology, Government Dental College, Raipur, Chhattisgarh, India; 2 Department of Oral and Maxillofacial Prosthodontics, All India Institute of Medical Sciences (AIIMS), Raipur, Chhattisgarh, India

**Keywords:** Titanium implants, oral corrosion mechanisms, biofilm-mediated degradation, titanium ion release

## Abstract

Titanium dental implants are widely used for their biocompatibility and mechanical strength but are vulnerable to corrosion within the oral environment. Corrosive processes such as galvanic, pitting, crevice and fretting corrosion are influenced by salivary pH, fluoride concentration, bacterial biofilms and mechanical forces. These factors can disrupt the protective oxide layer, leading to titanium ion release, local inflammation, peri-implantitis and potential implant failure. Therefore, it is of interest to review the mechanisms and clinical implications of titanium implant corrosion.

## Background:

Dental implants are commonly made from titanium and its alloys, which are used to restore missing teeth in partially and completely edentulous patients. The three most frequently used materials for these implants are: 1. Commercially Pure Titanium (CpTi): Renowned for its exceptional biocompatibility and resistance to corrosion 2. Titanium-Aluminum-Vanadium Alloy (Ti6Al4V): Enhanced strength and durability due to the addition of aluminum and vanadium 3. Titanium-Zirconium Alloy (TiZr): Offers superior corrosion resistance and osseointegration capabilities compared to Ti6Al4V [1]. "Despite titanium's chemical stability and passive nature, which forms a protective oxide layer, corrosion can still occur in implant applications due to the metal's exposure to the body's corrosive environment and repetitive stress. Corrosion is a process where a metal, in this case, titanium, reacts with its surroundings, leading to degradation or complete dissolution. This can happen even with titanium's protective oxide coating, especially under the constant and cyclic loads implants face in the human body [2]. Therefore, it is of interest to review the side effects of metal-based dental implantology treatment.

## Factors affecting corrosion of titanium implants/prosthesis: 

Multiple biological, chemical and mechanical agents participate in the corrosive action on titanium implants and components, amongst these factors, saliva, its composition, pH & buffering capacity, bacteria, infections, implant abutment interface and occlusal loads play a pivotal role as shown in Figure 1 (see PDF). Corrosion in titanium implants may occur as galvanic, pitting, crevice, fretting, or erosion types, often influenced by environmental factors. The classification and mechanisms of these corrosion types are summarized in Table 1 (see PDF).

## Saliva:

## Saliva as a weak electrolyte:

Saliva, being a weak electrolyte, facilitates electrochemical reactions that affect the stability of the titanium oxide layer. This oxide layer is critical for the corrosion resistance of titanium implants. Over time, continuous contact with saliva may result in the gradual dissolution of the oxide deposit, leaving the implant surface vulnerable to further degradation. Further, in the electrochemical reactions at the implant-saliva interface, electrons (e-) are attracted from saliva to the positively charged metal ions (M^+^) of titanium (Ti). This interaction occurs when the oxide layer on the titanium is damaged or compromised, initiating a localized electrochemical reaction. This electron exchange contributes to the corrosion of titanium as the exposed metal surface interacts with the ions present in the saliva [5].

## Ions in saliva:

Various ions from food and saliva, such as oxygen (O_2_), chlorine (Cl^-^), ferric ion (Fe^3+^) and cupric ion (Cu^2+^), interact with titanium surfaces. While these oxidizing species tend to reduce general corrosion by promoting the passive oxide layer, they may also contribute to localized corrosion under certain conditions. For instance, in crevices or fissures around the implant, these species can lead to crevice corrosion, which occurs in areas where oxygen access is limited. Chlorine ions (Cl^-^), in particular, are known to penetrate the oxide layer, causing localized damage and pitting corrosion [3].

## Fluoride concentration:

The fluoride content in dental products plays a major role in titanium corrosion. Fluoride ions, especially in high concentrations, can attack the protective oxide layer of titanium. In solutions with fluoride concentrations above 0.5% (5000 ppm), the corrosion resistance of titanium is significantly compromised, leading to pitting and localized corrosion. This is especially true in acidic conditions, as the fluoride ions enhance the dissolution of the oxide layer, increasing the risk of implant degradation. Acidic environments (low pH) exacerbate the effect of fluoride on titanium, as the oxide layer weakens in these conditions. Many dental fluoride gels and rinses, which are used for caries prevention, have pH levels as low as 3.5, further promoting corrosion in titanium-based dental implants or prosthetics. The combination of high fluoride concentration and low pH significantly increases the risk of corrosion [6].

## Chloride concentration:

Chloride ions (Cl^-^), commonly present in saliva, are known to exacerbate corrosion, particularly in confined spaces such as crevices. These ions can penetrate the outer oxide protective layer which causes pitting corrosion and weakens the implant surface. When saliva infiltrates between dental implants and implant-supported structures, it can create a galvanic cell an electrochemical setup that accelerates the corrosion process. This is especially problematic in modular implants or areas where different materials are used in close contact, as galvanic corrosion may develop between the metals [7].

## Salivary pH:

The pH of saliva directly influences the corrosion behavior of titanium. Under more acidic conditions, the titanium oxide layer is less stable and the metal becomes prone to active dissolution. Acidic environments, such as those caused by inflammation or bacterial activity, reduce the oxygen needed for the reformation of the oxide layer, thereby leading to increased ion leakage from the implant surface. As Duffo *et al.* observed, low pH levels in saliva (as seen in conditions like peri-implantitis with a pH of 5.2) result in significantly higher corrosion rates. In such chronic inflammatory conditions, titanium implants are particularly vulnerable to degradation, with the acidic environment accelerating the breakdown of the oxide layer [7].

## Bacteria:

Lipopolysaccharides from gram-negative bacteria can trigger inflammation in peri-implant tissues by affecting various cell types, including macrophages and osteoblasts. These lipopolysaccharides initiate corrosion on titanium dental implants by targeting and damaging the oxide layer, which exposes the titanium and allows ion exchange with saliva. Bacterial adherence to implants creates an acidic environment in two main ways: (i) Acidic Metabolic Products: Acid-producing bacteria like Streptococcus reduce pH and contribute to corrosion. (ii) Localized Crevice Corrosion: Biofilm formation leads to oxygen depletion in certain areas, reducing pH and causing corrosion and metal ion release [8].

## Infection:

Peri-implantitis disrupts the protective titanium dioxide layer on dental implants, leading to corrosion. Bacteria associated with peri-implantitis not only trigger inflammation but also cause electrochemical changes on the titanium surface, accelerating this corrosion. This breakdown releases titanium ions, which can worsen the inflammatory response. Research by Safioti *et al.* (2017) found that implants affected by peri-implantitis have higher levels of dissolved titanium in the surrounding plaque compared to healthy implants, linking titanium dissolution to the infection. This creates a harmful cycle where corrosion and inflammation feed into each other, potentially leading to implant failure [9].

## Implant-abutment interface:

The implant-abutment interface is a key site for titanium ion release due to mechanical wear, especially when different materials are used together. If a titanium implant is paired with a harder abutment like zirconia, the titanium may wear down; if the abutment is softer, it may degrade instead. This wear releases particles or ions into surrounding tissues, potentially causing inflammation or discoloration. The oral environment further promotes corrosion and when combined with wear (tribo-corrosion), it increases titanium ion release. These ions can trigger inflammatory responses; bone loss and compromise implant stability over time [8].

## Occlusal loads:

Micromotion in dental implants, especially under normal biting forces, can disrupt osseointegration if healing is incomplete or implant placement is suboptimal. This slight movement prevents proper bone fusion and may lead to implant loosening or failure. Additionally, micromotion contributes to corrosion, particularly fretting and crevice corrosion, by damaging the protective titanium oxide layer. Acidic by-products from oral bacteria further worsen this, promoting metal ion release (e.g., titanium) into surrounding tissues. This can trigger inflammation and peri-implantitis, weakening bone support. Over time, these mechanical and chemical effects compromise implant stability and longevity, increasing the risk of fracture or failure [8].

## Clinical significance of corrosion:

In comparison with CoCr & stainless steel, the corrosion resistance of Ti alloys is superior, however, when the corrosion occurs there is dissolution of Ti & its alloying components like aluminium, vanadium etc. which in turn generates localized and sometimes generalized host response. The cascade of biological events triggered by titanium corrosion ranges from mild tissue discoloration to severe inflammatory reactions, eventually leading to implant failure. These progressive host responses are summarized in Figure 2 (see PDF) [10].

## Tissue reaction in response to corrosion of titanium implants and components:

Corrosion of dental implants results in the leach out of titanium ions due to the electrochemical reaction in the oral environment, which leads to disruption in the titanium dioxide (TiO_2_) layer. This disruption triggers inflammatory responses in surrounding soft tissues (e.g., fibroblasts) and bone. The inflammation is driven by signaling factors that promote the differentiation and recruitment of osteoclasts, leading to increased bone resorption around the implant. When metallic particles or ions are released from dental implants, they can trigger a peri- implant inflammatory process through several mechanisms:

## Activation of phagocytic cells: 

Neutrophils and macrophages are activated, leading to an inflammatory response.

## Osteoblastic communication: 

The inflammatory process can stimulate communication pathways in osteoblastic cells.

## Microbial accumulation: 

The roughened surface of the degraded implant can promote microbial accumulation. There is increased expression of pro-inflammatory cytokines due to phagocytosis of Ti ions, which causes inflammation in tissue & bone resorption. This inflammation leads to decreases in pH in the affected area and causes microbial dysbiosis, leading to the production of bacterial acids. These acids further contribute to the degradation of titanium, negatively impacting its corrosion resistance [10].

## Inflammatory swelling and Pain:

Watterhehn *et al.* (1992) studied the effects of ionic release due to corrosion in dental implants, which can lead to pain and swelling, at tissues with infection [11].

## Bone loss and osteolysis:

The leaching of ions due to corrosion contributes to peri-implantitis and implant failure. The leached particles from the corrosion process are phagocytosed by macrophages, triggering an inflammatory response. This leads to the release of cytokines, which act as inflammatory mediators. These cytokines inhibit osteoblast production, enhancing the activity of bone- resorbing cells (osteoclasts), resulting in peripheral osteolysis and eventual loosening of the implant [12].

## Peri-implantitis:

The peri-implant microbiota, the host and Ti particles and ions may have a three-way relationship where each factor can affect the other.

[1] Microbiome triggers inflammation in host tissues and host factors influence microbial colonization.

[2] Titanium particle induces inflammation and host response in turn leads to titanium corrosion.

[3] Titanium affects the microbiome and microbial colonization can worsen titanium corrosion.

Together or independently, these factors sustain peri-implant inflammation.

## Fracture of dental implant:

Dental implant or prosthesis fractures are rare but can have serious clinical consequences. Such fractures are often linked to mechanical performance issues and the use of screw preload devices for securing abutments. Corrosion can significantly weaken both the material's resistance to repeated stress and its maximum load-bearing capacity, leading to potential structural failure. After four years of use, corrosion in end-osseous implant superstructures led to the release of metal ions into the surrounding tissues, which contributed to fatigue fractures [13]. On the other hand, environmental-induced cracking (EIC) and hydrogen embrittlement were investigated as factors contributing to lateral fractures of titanium implants in the oral environment [14]. For confirmed metal hypersensitivity, the definitive treatment is removal of the implant, though medical management with corticosteroids or atropine may be used in less severe cases. Zirconia and Polyetheretherketone (PEEK) are promising nonmetallic alternatives that demonstrate good biocompatibility and mechanical performance without triggering allergic responses [15]. Metal implants, despite their clinical importance, can trigger toxicity, irritation, and allergic reactions through corrosion and biological interaction. Future strategies should focus on advanced biomaterials, surface modification, and personalized implant selection to enhance long-term safety and compatibility [16].

## Conclusion:

Titanium dental implants, though widely recognized for their mechanical strength and biocompatibility, remain vulnerable to corrosion under oral conditions characterized by low pH, chloride and fluoride ions. These factors, particularly in crevices and microgaps, can compromise the oxide layer, leading to titanium particle release and potential peri-implant inflammation or failure. The degradation not only affects the structural performance of the implant but also interferes with tissue integration. However, corrosion risks can be minimized through optimized implant design, surface engineering and careful control of oral hygiene factors-especially pH and fluoride exposure. Advanced surface modifications, including anodic oxidation and biocompatible coatings, have shown significant promise in enhancing corrosion resistance and promoting osseointegration. Together, these strategies contribute to improved long-term implant stability and clinical success, reinforcing the importance of material science in modern dental practice.
